# Essential Oil of *Melaleuca ericifolia* Sm.: Chemical Composition, Enantiomeric Distribution, and Antimicrobial, Antioxidant, and Anticholinesterase Activities

**DOI:** 10.3390/molecules31193486

**Published:** 2026-09-30

**Authors:** Eduardo Valarezo, Alejandro Alvear-Coba, Fernando Seraquive-Paqui, Ximena Jaramillo-Fierro, James Calva, Miguel Angel Meneses

**Affiliations:** 1 Departamento de Química y Producción, Universidad Técnica Particular de Loja, Loja 1101608, Ecuador; xvjaramillo@utpl.edu.ec (X.J.-F.); jwcalva@utpl.edu.ec (J.C.); mameneses@utpl.edu.ec (M.A.M.); 2 Carrera de Ingeniería Química, Universidad Técnica Particular de Loja, Loja 1101608, Ecuador; laalvear1@utpl.edu.ec; 3 Carrera de Bioquímica y Farmacia, Universidad Técnica Particular de Loja, Loja 1101608, Ecuador; cfseraquive@utpl.edu.ec

**Keywords:** Ecuador, Loja, Myrtaceae, physical properties, GC-MS, GC-FID

## Abstract

*Melaleuca ericifolia* Sm., commonly known as paperbark, is a shrub or small tree native to Australia that has been introduced and cultivated in various regions worldwide due to its adaptability and diverse applications. In the present study, the chemical composition and biological activity of the essential oil isolated from this species are investigated. The essential oil was extracted through steam distillation. The qualitative and quantitative chemical composition of the essential oil was determined by gas chromatography. The enantiomeric distribution was determined using two enantioselective chromatographic columns. Antimicrobial activity was determined using the broth microdilution method against four Gram-negative bacilli, a Gram-positive bacillus, three Gram-positive cocci, one fungus and one yeast. The antioxidant activity was determined using ABTS and DPPH assays. Anticholinesterase activity was determined using a spectrophotometric method. The forty-seven compounds identified accounted for 99.74% of the overall composition. Oxygenated monoterpenes were the most representative group in terms of number of compounds (fourteen) and in terms of relative abundance (72.50%). The most abundant constituents were 1,8-cineole (61.85 ± 1.46%), limonene (11.23 ± 0.16%), *α*-terpineol (7.99 ± 0.10%), *α*-pinene (4.97 ± 0.17%) and *β*-pinene (3.71 ± 0.17%). Five pairs of enantiomers were identified in the essential oil from leaves of *Melaleuca ericifolia*. The essential oil presented MICs of 2000 and 4000 μg/mL against *Candida albicans* and *Aspergillus niger*, respectively. The antioxidant activity was weak with a SC_50_ of 2 mg/mL in the ABTS assay. The essential oil exhibited moderate anticholinesterase activity, with an IC_50_ of 73.6 ± 1.0 µg/mL. This study provides the first comprehensive analysis of the chemical composition and biological activities of the essential oil isolated from leaves of *Melaleuca ericifolia* Sm. cultivated in the Americas. Furthermore, the enantiomeric distribution and antioxidant and acetylcholinesterase inhibitory activities of this essential oil are reported for the first time.

## 1. Introduction

Essential oils (EOs) are complex mixtures of volatile secondary metabolites produced in various plant organs, including leaves, flowers, fruits, seeds, stems, roots, bark, and wood. These compounds are synthesized and accumulated in specialized secretory structures, such as secretory cells, cavities, ducts, and glandular trichomes. These natural products have been traditionally used for medicinal, culinary, and aromatic purposes across different cultures. EOs have been widely used for antiparasitic, bactericidal, cosmetic, fungicidal, insecticidal, medicinal and virucidal applications [1]. In recent decades, the growing interest in natural products has stimulated extensive research on essential oils, highlighting their chemical diversity and biological properties, as well as their importance in the pharmaceutical, cosmetic, food, and agricultural industries [2]. EOs are found in species of many botanical families and give plant species the status of aromatic plants [3]. Among the families with the most aromatic plants are Apiaceae, Asteraceae, Burseraceae, Cupressaceae, Lamiaceae, Lauraceae, Leguminosae, Myrtaceae, Pinaceae, Piperaceae, Rutaceae and Zingiberaceae [4].

Myrtaceae Juss. is a family of evergreen, aromatic, shrubby, and arboreal dicotyledonous plants belonging to the order Myrtales. The Myrtaceae family comprises approximately 127 genera and 6547 species [5]. Myrtaceae are widely distributed throughout the tropical and subtropical regions of the Americas, Africa, Asia, and Australia. They thrive in diverse terrestrial ecosystems, except for arid and semi-arid zones [6]. Most members of the Myrtaceae family have high economic value due to the presence of species with edible fruits and raw materials for obtaining essential oils, spices, and timber. Likewise, several species are cultivated for their ornamental value [7]. Members of this family are characterized by the presence of secretory cavities rich in essential oils, as well as by their aromatic leaves and economic importance. EOs of several genera such as *Eucalyptus*, *Eugenia*, *Leptospermum*, *Melaleuca*, *Myrtus* and *Pimenta* have been studied due to their phytochemical composition and bioactive properties [4].

Within Myrtaceae, the genus *Melaleuca* L. includes 391 species native to Australia [8], although many species have been introduced and cultivated in other regions, such as the southern parts of North America, southeastern America, Southeast Asia, southern Africa and Europe, for ornamental, medicinal, and industrial purposes [9]. The name *Melaleuca* originates from the Greek words *melas*, meaning black or dark, and *leucon*, meaning white. This designation refers to the contrast observed in the first described species, *M. leucadendra*, which is characterized by its white branches and a dark trunk that is often blackened because of fire exposure [10]. Plants of this genus are commonly known as paperbark trees or tea trees and are characterized as evergreen shrubs or small trees with aromatic leaves rich in essential oils. The essential oils obtained from *Melaleuca* species are used in traditional medicine and modern pharmacology because of their antimicrobial, anti-inflammatory, antioxidant, and insecticidal activities [11].

*Melaleuca ericifolia* Sm., commonly known as paperbark or swamp paperbark, is a shrub or small tree reaching 1–8 m in height. The bark of this tree is layered and papery, providing a texture that peels away in strips, hence the name paperbark [12]. Its branchlets are glabrescent to slightly lanuginose, and the leaves are alternate or arranged in threes, very narrow and linear to elliptic, measuring 5–18 mm long, with three longitudinal veins and abundant essential oil glands. The inflorescences are spicate or capitate, pseudoterminal, and composed of 10–40 flowers with deciduous petals and white or cream stamens grouped in bundles. The species produces small fruits with sepaline teeth and numerous seeds. It occurs naturally in coastal and subcoastal regions of southeastern Australia, including New South Wales, Victoria, and Tasmania, where it grows in moist habitats such as heathlands, swamp margins, wet sclerophyll forests, streamlines, coastal dunes, and sandy or alluvial soils [10,13]. Due to its adaptability, *M. ericifolia* has also been cultivated in other regions of the world with similar climatic conditions. In Ecuador, this species has the status of an introduced species.

Although most species of the genus *Melaleuca* possess essential oils, efforts of researchers have been concentrated on studying the most common species, *Melaleuca alternifolia* [14,15], and despite the economic and biological relevance of the genus *Melaleuca*, there is still limited information regarding the physicochemical characteristics, chemical variability, and biological activities of the essential oils of some species, particularly those cultivated outside their native habitats, among them *M. ericifolia*. Therefore, further studies are needed to explore the chemical composition and bioactive potential of this species. This fact motivated the authors to carry out this study with the aim of isolating the essential oil from the leaves of *Melaleuca ericifolia* and characterizing its physical properties and chemical profile. In addition, this research evaluates its biological potential through antimicrobial, antifungal, antioxidant, and anticholinesterase assays. The working hypothesis is that the essential oil of this species contains characteristic terpenoid compounds typical of the genus *Melaleuca* [10] and exhibits significant biological activities that support its potential application in pharmaceutical, cosmetic, and food industries.

## 2. Results

### 2.1. Physical Properties of Essential Oil

The essential oil from leaves of cultivated *M. ericifolia* was presented as a dark moderate yellow, unctuous liquid less dense than water. Table 1 shows the mean values of the physical properties of essential oil.

### 2.2. Chemical Composition of Essential Oil

Using gas chromatography coupled with mass spectrometry (GC-MS) and gas chromatography equipped with a flame ionization detector (GC-FID), forty-seven compounds were identified in the essential oil isolated from the leaves of *Melaleuca ericifolia*. Table 2 summarizes the compound number (CN) assigned according to their elution order, retention time (RT) of elution, calculated retention indices (RIC), literature retention indices (RIR), relative abundance (%) together with standard deviation (SD), chemical classification (GC), molecular formula (CF), and monoisotopic mass (MM) of the identified constituents. The compounds identified accounted for 99.74% of the overall composition. These constituents were grouped into five categories based on their carbon skeleton (monoterpenes with 10 carbons and sesquiterpenes with 15 carbons), the presence or absence of oxygenated functional groups, and the presence of non-terpenic compounds. Among them, oxygenated monoterpenes (OM) represented the predominant fraction in both the number of compounds (fourteen) and relative abundance, accounting for 72.50% of the total oil, followed by monoterpene hydrocarbons (MH) with 22.56%. In contrast, sesquiterpene hydrocarbons (SH), oxygenated sesquiterpenes (OS), and other compounds (OC) were found in much lower amounts, representing 1.39%, 2.45%, and 0.84%, respectively.

The most abundant constituents (>5%) were 1,8-cineole (CN: 10; CF: C_10_H_18_O), classified as an oxygenated monoterpene, with 61.85 ± 1.46%; *α*-terpineol (CN: **23**) with 7.99 ± 0.10%; and limonene (CN: **9**; CF: C_10_H_16_), a monoterpene hydrocarbon, with 11.23 ± 0.16%. Other noteworthy compounds, although present in lower concentrations, included *α*-pinene (CN: **2**) and *β*-pinene (CN: **5**), with relative abundances of 4.97 ± 0.17% and 3.71 ± 0.17%, respectively.

### 2.3. Enantiomeric Distribution

Using MEGA-DEX DET Beta and MEGA-DEX DAC Beta enantioselective stationary phase columns, five pairs of enantiomers were separated in the EO from leaves of *M. ericifolia*. Table 3 shows the retention time (RT), retention index (RI), enantiomeric distribution (ED) and enantiomeric excess (e.e.) for each pair of compounds. Only one limonene enantiomer was detected, although it could not be determined which one. Of the separated enantiomers, (−)-(1S,5S)-*β*-pinene was the most abundant with an enantiomeric excess of 90.14%, and *α*-terpineol was determined in both columns.

### 2.4. Antibacterial Activity

The antibacterial capacity of the EO of *M. ericifolia* was tested against four Gram-negative bacilli, one Gram-positive bacillus and three Gram-positive cocci. Table 4 shows the minimum inhibitory concentration (MIC) values of the EO and the positive and negative controls. It was not possible to determine the MIC value against any bacteria at the maximum concentration tested (4000 µg/mL). All microorganisms showed normal growth in the negative control.

### 2.5. Antifungal Activity

The EO of *Melaleuca ericifolia* was tested against a fungus and a yeast. Table 5 shows the values for the MIC of the EO and the positive and negative controls. The essential oil showed weak activity against *Candida albicans* with a MIC of 2000 µg/mL.

### 2.6. Antioxidant Activity

The antioxidant capacity of the essential oil was determined by two methods: ABTS and DPPH. Table 6 shows the half-maximal scavenging capacity (SC_50_) values expressed in µg/mL of the EO and the positive control, and the antioxidant capacity in µM/g for trolox equivalent antioxidant capacity (TEAC) values.

### 2.7. Anticholinesterase Activity

**Figure 1:**
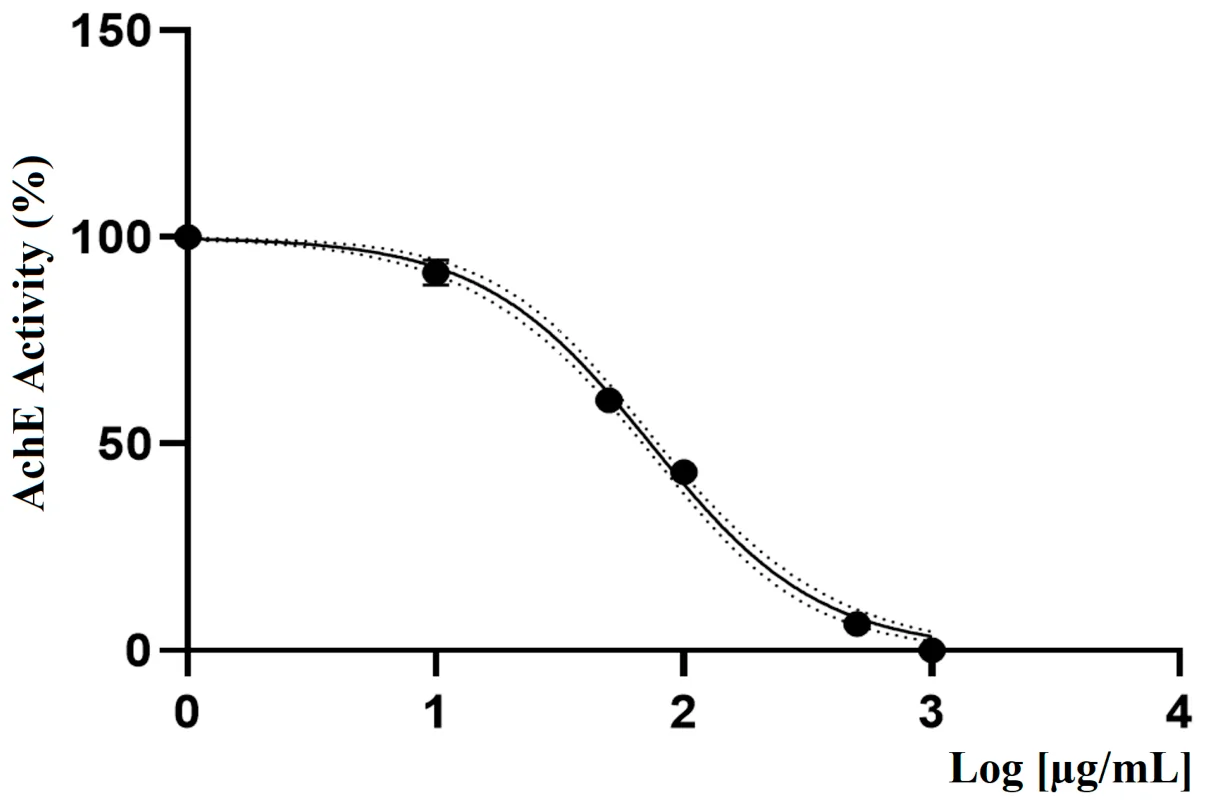
Anticholinesterase activity of essential oil from leaves of *Melaleuca ericifolia*.

Regarding the anti-enzymatic activity, the EO from leaves of *Melaleuca ericifolia* exhibited a half-maximal inhibitory concentration (IC_50_) value of 73.6 ± 1.0 µg/mL. The donepezil used as the positive control had an IC_50_ value of 12.4 ± 1.4 µg/mL. Figure 1 shows the reaction rate curve for anticholinesterase (anti-AChE) activity.

## 3. Discussion

The physical properties of EO, including color, density, refractive index, and specific optical rotation, are due to its chemical composition and relative abundance of its volatile constituents. The EO obtained in this study exhibited a dark khaki color (RGB: 191, 184, 118, Table 1). Besides serving as a descriptive characteristic, color represents an important quality attribute because it contributes to product standardization and influences consumer acceptance, particularly when essential oils are incorporated into food, cosmetic, or personal care formulations [16]. As expected for hydrophobic mixtures rich in terpenoids, the density of the essential oil was lower than that of water, a characteristic that also facilitates phase separation during hydrodistillation. The essential oil extracted from *M. ericifolia* leaves presented a density of 0.9221 ± 0.0041 g/cm^3^. It was not possible to find previous reports on the density of the EO of *M. ericifolia*. Studies on essential oils of the genus *Melaleuca* have found that the density of essential oils of this genus is usually lower than that of water and varies depending on the species. In *Melaleuca alternifolia* (tea tree), a density of 0.9048 ± 0.0015 g/mL has been determined [17]; the EO of *M. cajuputi* presented a density of 0.9086 ± 0.0002 g/mL, and for *M. armillaris*, a density of 0.9053 ± 0.0045 g/cm^3^ has been reported. There are also variations in density depending on the collection site, which implies variation in chemical composition. For *M. viridiflora*, relative density values between 0.830 and 0.838 were reported for oil extracted from species in Papua, Indonesia [18], and 0.8764 (Maryborough) and 0.8800 (Brisbane) for EO from Australia [19].

The refractive index is an important physicochemical characteristic of essential oils, as it can provide insight into their chemical composition. Its magnitude is determined by both the optical properties of the individual compounds and their relative abundance in the oil [20]. The essential oil obtained from *M. ericifolia* leaves exhibited a refractive index of 1.4650 ± 0.0001. This result is consistent with its monoterpene-rich composition, particularly the presence of 1,8-cineole, limonene, and *α*-pinene, compounds whose reported refractive index ranges are 1.448–1.490, 1.470–1.485, and 1.453–1.5053, respectively [21].

The essential oil of *M. ericifolia* exhibited a slightly positive optical rotation of +3.8 ± 0.10°, indicating a relatively low net optical activity. This parameter reflects the combined contribution of chiral constituents and, importantly, their enantiomeric distribution within the oil. The modest positive value observed here may be partly related to the predominance of 1,8-cineole, an achiral constituent that is not expected to contribute substantially to the overall optical rotation. Although Fujita and Yasuji [22] reported an optical rotation of +5.55° for 1,8-cineole, current specifications for the pure compound indicate values close to zero (−0.3° to +0.3° at 20 °C and 589 nm). Consequently, the optical activity of *M. ericifolia* essential oil is more plausibly associated with minor or less abundant chiral constituents. Limonene is particularly relevant in this context because its enantiomers display strong but opposite specific rotations, approximately +100° for (+)-limonene and −100° for (−)-limonene [21]. Thus, even at relatively low concentrations, variations in the enantiomeric ratios of limonene and other optically active terpenes may influence the net rotation of the oil. The measured value should therefore be interpreted as the cumulative outcome of the enantiomeric composition of the essential oil rather than as a property attributable to any single constituent.

Species of the genus *Melaleuca* are characterized by considerable chemical diversity in their essential oils, which comprise a complex mixture of mono- and sesquiterpenoids, together with less abundant phenylpropanoid derivatives. Frequently reported monoterpenoids include terpinen-4-ol, terpinolene, *p*-cymene, *α*-terpineol, 1,8-cineole, *α*-pinene, and *α*-terpinene, whereas *β*-caryophyllene, ledol, (*E*)-nerolidol, and viridiflorol are among the representative sesquiterpenoids. Phenylpropanoids, including methyl eugenol and methyl isoeugenol, have also been identified in oils from *Melaleuca* species [23,24]. Among members of this genus, *Melaleuca alternifolia* is by far the most extensively investigated species, primarily because its essential oil is commercially recognized as tea tree oil. Terpinen-4-ol generally constitutes its predominant component, accompanied by substantial proportions of *α*-terpinene, *γ*-terpinene, *α*-terpineol, and 1,8-cineole (eucalyptol). Nevertheless, the qualitative and quantitative composition of the oil is not constant, as the relative abundance of these constituents may vary considerably according to geographical origin, environmental conditions, plant material, and other factors influencing secondary metabolism [14,25].

The qualitative and quantitative analysis of EO from leaves of *M. ericifolia* revealed a complex chemical profile comprising 47 identified components, accounting for 99.74% of the total composition. The constituents were categorized into two major groups based on their biosynthetic origin and structural features: monoterpene hydrocarbons (22.56%) and oxygenated monoterpenes (72.50%). The most abundant constituent was 1,8-cineole (61.85 ± 1.46%), followed by limonene (11.23 ± 0.16%) and *α*-terpineol (7.99 ± 0.10%).

There are few studies in the literature on the chemical composition of *M. ericifolia* essential oil. These studies show that there is no single major compound for this oil; at least three distinct chemical profiles exist, dominated by methyl eugenol, 1,8-cineole, or linalool. Farag et al. [26] in 2004 reported that the essential oil of *M. ericifolia* collected in Kerdasa, Giza, Egypt, is rich in methyl eugenol (96.84%), while Khedhri et al. [27] in 2024 reported 1,8-cineole (83.55–92.35%) as the major compound in EO from Tunisia, and Vázquez et al. [28] reported in 2023 that linalool (38.19%) is the major component of a commercial EO (Nature’s Gift, Madison, TN, USA). Furthermore, the chemical composition of *M. ericifolia* EO exhibits geographical variability, particularly in the relative abundance of 1,8-cineole and linalool. Brophy and Doran [29], who examined populations across the natural distribution of the species in southeastern Australia, reported a clear latitudinal trend; oils from northern populations in New South Wales were generally characterized by high linalool levels, whereas those from southern Victoria and Tasmania were richer in 1,8-cineole. A specimen collected at Hawks Nest, New South Wales, contained 60% linalool and 16% 1,8-cineole, while the southernmost populations showed an average 1,8-cineole content of 34.5% and only approximately 3% linalool.

The predominance of 1,8-cineole is not restricted to a single *Melaleuca* species, as this oxygenated monoterpene has been reported as a major constituent in the essential oils of several members of the genus. For instance, the leaf essential oil of *M. atroviridis* contains 73.2% 1,8-cineole, representing its most abundant constituent [30]. An even higher proportion has been documented for a 1,8-cineole-rich chemotype of *M. teretifolia*, in which this compound accounts for 84.0% of the total essential oil [31]. Similarly, *M. fulgens* has been reported to contain 56.9% 1,8-cineole [32]. Considerable intraspecific variation has also been observed in *M. quinquenervia*, with concentrations reaching up to 76% in some samples, supporting the occurrence of distinct 1,8-cineole-rich chemotypes within this species [24]. In contrast, *M. leucadendron* contains a comparatively low proportion of 1,8-cineole (19.9%); nevertheless, it remains the main component of its volatile fraction [33]. Collectively, these findings highlight the widespread occurrence of 1,8-cineole across the genus *Melaleuca* and demonstrate that its relative abundance can vary substantially among species and chemotypes.

The enantiomeric analysis of the EO of *M. ericifolia* revealed stereochemical selectivity among the chiral monoterpenes, with enantiomeric excesses (e.e.) of 63.66% for *α*-pinene, 90.14% for *β*-pinene, 100% for limonene, 53.23% for *α*-terpineol, and 51.15% for linalool. In particular, (−)-(1*S*,5*S*)-*β*-pinene predominated (95.07%), whereas limonene was detected as a single enantiomeric form (ED = 100%), indicating strong stereoselectivity in their biosynthesis. However, it was not possible to conclusively assign the absolute configuration of the detected limonene enantiomer, as single-enantiomer reference standards were unavailable and no co-injection assays were performed. Wong et al. [34] highlight the importance of characterizing the enantiomeric distribution of terpenes in essential oils, as it may provide a more specific chemical fingerprint than conventional compositional analysis for assessing their origin, quality, and authenticity. The enantiomeric distribution of the EO of *M. ericifolia* has not been reported before; however, in 57 samples of *M. alternifolia* essential oil obtained from Australian plantations, the authors observed consistent enantiomeric ratios for terpinen-4-ol [68.5% (+):31.5% (−)] and *α*-terpineol [74.2% (+):25.8% (−)], whereas several commercial samples showed substantial deviations from these characteristic patterns [34]. The relative abundance of individual enantiomers is closely associated with the stereoselective biosynthetic pathways of the plant, and their determination by enantioselective gas chromatography can provide valuable chemical markers for establishing species-specific profiles and detecting potential adulteration or the incorporation of compounds from different origins. Therefore, enantiomeric analysis complements conventional chemical characterization and provides additional information of considerable value for the authentication, standardization, and quality control of *Melaleuca* essential oils.

The EO of *Melaleuca ericifolia* evaluated in the present study showed no antibacterial activity up to the maximum concentration tested (4000 µg/mL) against Gram-negative (*C. jejuni*, *E. coli* O157, *P. aeruginosa*, and *S. enterica* serovar Typhimurium) and Gram-positive bacteria (*L. monocytogenes*, *E. faecalis*, *E. faecium*, and *S. aureus*). These findings contrast with previous reports showing strain-dependent antibacterial activity for this species. Marinković et al. [35] reported MIC values of 0.65–23.35 mg/mL for the EO of *M. ericifolia* against facultative and obligate endodontic anaerobes. Klūga et al. [36] reported that the EO of *Melaleuca ericifolia,* rich in linalool (47.5%), showed antibacterial activity against pathogenic microorganisms of freshwater fish, with MIC values ranging from 1.56 to 50 µL/mL. The highest susceptibility was observed for *Enterococcus aquimarinus* (MIC = 1.56 µL/mL), followed by *Aerococcus* spp., *Aeromonas* spp., *A. bestiarum*, *Enterococcus faecium*, *E. moravensis*, *Yersinia enterocolitica*, and *Yersinia* spp., which exhibited MIC values of 6.25 µL/mL. Intermediate susceptibility (MIC = 12.5 µL/mL) was recorded for *Aeromonas salmonicida*, *Escherichia vulgaris*, *Pseudomonas fluorescens*, *P. gessardii*, *P. ludensis*, *P. proteolitica*, *Shewanella baltica*, *Yersinia ruckeri*, and *Vagococcus* spp. In contrast, *Aerococcus viridans* (25 µL/mL) and particularly *Pseudomonas frederiksbergensis* (50 µL/mL) were less susceptible to the essential oil. Farag et al. [26] also reported antimicrobial activity of the EO of *M. ericifolia*, particularly against *Bacillus subtilis*. However, their oil was characterized by a high methyl eugenol content (96.84%). In contrast, the EO analyzed in the present study was dominated by 1,8-cineole (61.85%), indicating a markedly different chemical profile. Therefore, the comparatively low antibacterial activity observed here may reflect differences in chemotype and the relative abundance and interactions of bioactive constituents, as well as variations in bacterial strains and experimental conditions. Overall, these findings emphasize that the antibacterial potential of *M. ericifolia* EO cannot be generalized at the species level but should be interpreted according to its specific chemical profile and the susceptibility of the target microorganism.

The essential oil of *M. ericifolia* exhibited relatively weak antifungal activity, with MIC values of 2000 µg/mL against *Candida albicans* ATCC 10231 and 4000 µg/mL against *Aspergillus niger* ATCC 6275, indicating greater susceptibility of the yeast than the filamentous fungus. In the literature, only one study of the antifungal activity of this essential oil was found, that of Farag et al. in 2004 [26]. The study was carried out using the inhibition zone method against *Aspergillus niger* and *Candida albicans*. The results established that the essential oil of this species exhibited the highest inhibitory effects against Aspergillus niger [26]. At the genus level, comparatively stronger antifungal activity has been documented for other *Melaleuca* species. For instance, the EO of *M. alternifolia* has shown inhibitory effects against filamentous fungi, including *Penicillium italicum* and *P. digitatum* [37]. Similarly, the EO of *M. cajuputi* exhibited pronounced antifungal activity against toxigenic *Aspergillus flavus*, suppressing both fungal growth and aflatoxin B1 production at relatively low concentrations [38]. Mechanistic analyses further revealed alterations in ergosterol biosynthesis, increased leakage of intracellular constituents, and disruption of mitochondrial membrane potential, suggesting that the antifungal action of *Melaleuca* EOs may involve multiple cellular targets [38]. Such differences in antifungal potency among species may be partly associated with variations in their chemical profiles. Notably, the EO of *M. cajuputi* investigated by Chaudhari et al. [38] was dominated by *α*-pinene (49.24%), whereas the EO examined in the present study was characterized by a predominance of 1,8-cineole. These findings indicate that antifungal activity within the genus *Melaleuca* should not be inferred solely from taxonomic relatedness; rather, it appears to be influenced by the qualitative and quantitative composition of each EO, including potential interactions among its major and minor constituents [39].

The EO of *M. ericifolia* exhibited weak radical-scavenging activity, with SC_50_ values of 2001.1 ± 1.5 µg/mL in the ABTS assay and 7657.5 ± 16.1 µg/mL in the DPPH assay. To the best of our knowledge, this is the first study of antioxidant activity for the EO of this species, limiting direct comparisons with previous studies. Nevertheless, substantially stronger antioxidant effects have been documented for other members of the genus *Melaleuca*. Zhang et al. [37] reported an EC_50_ of 48.35 µg/mL for DPPH radical scavenging by *M. alternifolia* EO, together with an EC_50_ of 43.71 µg/mL for hydroxyl radical scavenging and an IC_50_ of 135.9 µg/mL in the TBARS assay. Similarly, *M. cajuputi* EO displayed DPPH and ABTS radical-scavenging activities with IC_50_ values of 3.16 and 4.29 µL/mL, respectively [38]. The differences among *Melaleuca* EOs may be partly attributable to variations in their chemical profiles and to potential synergistic or antagonistic interactions among their constituents. For example, monoterpenes such as *α*-terpinene and terpinolene have been associated with the antioxidant properties of *M. alternifolia* EO. Furthermore, the approximately four-fold difference between the ABTS and DPPH SC_50_ values observed for *M. ericifolia* highlights the assay-dependent nature of antioxidant responses, which may be influenced by differences in radical chemistry, reaction mechanisms, and constituent accessibility. Overall, these findings indicate limited direct radical-scavenging capacity of the EO of *Melaleuca ericifolia* under the conditions tested; however, they do not exclude the possibility of antioxidant effects mediated through mechanisms not assessed by the ABTS and DPPH assays.

According to the potency criteria proposed by Santos et al. [40], which classify AChE inhibition as high (IC_50_ < 20 µg/mL), moderate (20 < IC_50_ < 200 µg/mL), or low (200 < IC_50_ < 1000 µg/mL), the EO of *M. ericifolia* exhibited moderate acetylcholinesterase (AChE) inhibitory activity, with an IC_50_ of 73.61 ± 1.0 µg/mL. It was not possible to find previous studies on the AChE inhibitory activity of *M. ericifolia* EO. Nevertheless, anticholinesterase activity has been documented for other members of the genus *Melaleuca*. *M. alternifolia* EO showed an IC_50_ of 51.2 µg/mL [41], while commercial tea tree oils exhibited IC_50_ values of 0.05–0.11 µL/mL [42]; complete AChE inhibition has also been reported at 100 µL/mL in an *Anisakis simplex*-based assay [43]. In comparison, *M. cajuputi* EO produced 21.18% AChE inhibition under the conditions evaluated by Petrachaianan et al. [44]. Differences in inhibitory potency among these oils may be partly associated with their distinct chemical profiles. For instance, the essential oil of *M. alternifolia* investigated by Miyazawa et al. [41] was dominated by terpinen-4-ol (35.6%), *γ*-terpinene (19.4%), and *α*-terpinene (8.3%), whereas the essential oil of *M. ericifolia* evaluated here was characterized by a predominance of 1,8-cineole. Although the contribution of individual constituents cannot be established from the present data, essential oil terpenoids may modulate AChE activity through different modes of enzyme inhibition, and the overall response may arise from additive, synergistic, or antagonistic interactions among multiple constituents [45]. Moreover, the reported AChE-inhibitory properties of individual monoterpenes, including *α*-pinene [46], support the potential contribution of both major and minor constituents to the moderate inhibitory activity observed for *M. ericifolia* EO.

## 4. Materials and Methods

### 4.1. Materials

The aliphatic hydrocarbons employed for constructing the GC-FID calibration curve were obtained from ChemService (West Chester, PA, USA), while helium was supplied by INDURA (Quito, Ecuador). For antimicrobial assays, Sabouraud dextrose broth, fluid thioglycollate medium, Mueller–Hinton broth, and Mueller–Hinton II broth were purchased from DIPCO (Quito, Ecuador). Phosphate-buffered saline (PBS) and tris hydrochloride (Tris-HCl) were acquired from Sigma-Aldrich (St. Louis, MO, USA).

For the evaluation of bioactivity, the reagents 2,2-diphenyl-1-picrylhydrazyl (DPPH), 2,2′-azinobis(3-ethylbenzothiazoline-6-sulfonic acid) (ABTS), 5,5′-dithiobis(2-nitrobenzoic acid) (DTNB), acetylcholinesterase (AChE), acetylthiocholine (AcSCh), butylated hydroxytoluene (BHT), dichloromethane (DCM), donepezil, dimethyl sulfoxide (DMSO), methanol (MeOH), magnesium chloride hexahydrate, anhydrous sodium sulfate, and trolox were also purchased from Sigma-Aldrich. All chemicals were of analytical grade and were used as received, without further purification.

### 4.2. Plant Material

Leaves of cultivated *Melaleuca ericifolia* were harvested in the vicinity of the Guayllabamba parish, located in the Quito canton of Pichincha province. The sampling site corresponded to a valley situated at 0°04′43″ S and 78°20′59″ W, at an altitude of 2171 m above sea level. After collection, the plant material was carefully stored and transported in sealed plastic containers. During both the collection and transportation processes, the environmental conditions were maintained at a pressure of approximately 79 kPa and a temperature ranging between 18 and 20 °C.

### 4.3. Postharvest Treatments

The leaves collected were immediately subjected to postharvest treatment. This treatment involved the removal of foreign material or deteriorated leaf samples.

### 4.4. Essential Oil Isolation

The whole *Melaleuca* leaves were extracted by steam distillation using a distiller (200 L, local construction). The process began by placing the fresh leaves in the distiller and passing steam through them; the steam was previously obtained in a boiler. Extraction was conducted for 3 h, from the collection of the first drop of distillate. The resulting vapor, containing the EO and water, was condensed, and the EO was separated by decantation. The EO was dried with anhydrous sodium sulfate. The dried EO was then stored at 4 °C in sealed amber vials.

### 4.5. Determination of the Physical Properties of the Essential Oil

Three objective physical parameters of the EO were evaluated, namely density, refractive index and optical rotation, together with one subjective characteristic, color. The density was determined according to the AFNOR NF T 75-111 standard, which is equivalent to ISO 279:1998 [47], using an analytical balance (Mettler AC 100, Mettler Toledo, Columbus, OH, USA). The refractive index was measured following the AFNOR NF T 75-112 protocol, comparable to ISO 280:1998 [48], with an ABBE refractometer (BOECO, Hamburg, Germany). Optical rotation was assessed in accordance with ISO 592:1998 [49] employing an automatic polarimeter (Mrc-P810, MRC, Holon, Israel). In addition, the color of the EO, considered a subjective attribute, was determined through digital analysis using the PineTools platform (website https://pinetools.com/, accessed on 13 December 2025), where an image of the sample placed on a white background was uploaded. All determinations were performed under controlled conditions at a constant temperature of 20 °C.

### 4.6. Identification of Essential Oil Compounds

The chemical constituents of EO isolated from leaves of *M. ericifolia* were characterised by gas chromatography (GC) using a TRACE™ 1310 gas chromatograph (Thermo Scientific, Waltham, MA, USA), provided with Chromeleon 7.2 software for data acquisition and supported by the NIST 17 and ADMIS 2.7 mass spectral libraries. Quantitative determination was performed with a flame ionisation detector (GC–FID), whereas qualitative identification was carried out using a quadrupole mass spectrometer (GC–MS) ISQ 7000 (Thermo Scientific, Waltham, MA, USA). In both analyses, an AI 1310 autosampler (Thermo Scientific, Waltham, MA, USA) was employed.

Chromatographic separation was achieved using a capillary column (30 m × 0.25 mm i.d., 0.25 µm film thickness) with a stationary phase consisting of 5% phenyl–95% dimethylpolysiloxane. Prior to injection, the sample was diluted in dichloromethane (1:100, *v*/*v*). A total of 1 µL of the solution was introduced in split mode with a ratio of 1:50. Helium was used as the carrier gas. For GC–FID analysis, the flow rate was adjusted to 1.0 mL/min, corresponding to an average linear velocity of 25 cm/s, while for GC–MS the flow rate was set at 0.9 mL/min, with a linear velocity of 34 cm/s. The injector and detector temperatures were maintained at 230 °C. The oven temperature programme was the same for both methods: an initial temperature of 50 °C held for 3 min, followed by a ramp from 50 to 230 °C at 3 °C/min, and a final hold at 230 °C for 3 min, resulting in a total analysis time of 66 min.

Relative compound abundances were estimated from the peak areas obtained by GC–FID, without the application of response correction factors. Mass spectrometric detection was conducted in electron impact (EI) mode at 70 eV, with a scanning range of 40–350 *m*/*z*, a scan speed of 2 scans/s, and an electron multiplier voltage of 1600 V. Retention indices (RI) were calculated using Equation (1) [50] based on a homologous series of *n*-alkanes analysed under identical chromatographic conditions, as previously described.

(1) $$RI=100C+100\frac{\left(t_{R}\left(x\right)-t_{R}\left(n\right)\right)}{\left(t_{R}\left(n+1\right)-t_{R}\left(n\right)\right)}$$

where *n* is the number of carbon atoms of the *n*-alkane eluting immediately before the compound of interest, $t_{R}\left(x\right)$ is the retention time of the analyzed compound, and $t_{R}\left(n\right)$ and $t_{R}\left(n+1\right)$ correspond to the retention times of the *n*-alkanes eluting immediately before and after the compound, respectively.

Compounds were identified by comparing their RI and mass spectra with published data [51].

### 4.7. Enantioselective Analysis

The enantiomeric composition of the essential oil (EO) obtained from *Melaleuca ericifolia* leaves was analysed using the same gas chromatograph, injector, and mass spectrometric detector described for the qualitative analysis. To evaluate the enantiomeric distribution, two chiral capillary columns (30 m × 0.25 mm × 0.25 μm) were employed. One column, MEGA-DEX DET-Beta (Mega, Legnano, Italy), was coated with a diethyl tert-butyldimethylsilyl-*β*-cyclodextrin stationary phase, whereas the second, MEGA-DEX DAC-Beta (Mega, Legnano, Italy), contained a diacetyl tert-butyldimethylsilyl-*β*-cyclodextrin stationary phase.

The split ratio, carrier gas, flow rate, injector and detector temperatures, temperature programme, and mass spectrometry conditions were identical to those previously described for qualitative GC–MS analysis. The elution sequence of each pair of enantiomers was established according to the technical information provided for the columns. The enantiomeric excess (e.e.) was calculated as the difference between the relative percentage of the predominant enantiomer and that of the corresponding minor enantiomer.

### 4.8. Antimicrobial Activity

#### 4.8.1. Determination of Antibacterial Activity

The antibacterial activity of the essential oil isolated from *Melaleuca ericifolia* was evaluated against a panel of reference microorganisms. Gram-negative bacilli included *Campylobacter jejuni* (ATCC 33560), *Escherichia coli* O157:H7 (ATCC 43888), *Pseudomonas aeruginosa* (ATCC 10145) and *Salmonella enterica* subs enterica serovar *Typhimurium* WDCM 00031, derived from ATCC 14028. Gram-positive cocci comprised *Enterococcus faecalis* (ATCC 19433), *Enterococcus faecium* (ATCC 27270), and *Staphylococcus aureus* (ATCC 25923), while the Gram-positive bacillus tested was Listeria monocytogenes.

The antibacterial assay was carried out following the protocol previously described by Valarezo et al. [52]. Briefly, the test was performed in 96-well microplates using two-fold serial dilutions of the EO, with concentrations ranging from 4000 to 15.62 µg/mL. For *Campylobacter jejuni*, the tested range was 4000 to 31.25 µg/mL [53]. Ampicillin was used as the positive control for Gram-positive cocci, whereas ciprofloxacin served as the positive control for both the Gram-positive bacillus and Gram-negative bacilli. Dimethyl sulfoxide (DMSO) at 5% was employed as the negative control, and the highest EO concentration evaluated was 4000 µg/mL. Antibacterial activity was expressed as the minimum inhibitory concentration (MIC), defined as the lowest EO concentration capable of preventing visible microbial growth after incubation.

#### 4.8.2. Determination of Antifungal Activity

The antifungal activity of the EO isolated from *Melaleuca ericifolia* was determined according to the method described by Valarezo et al. (2021) [52]. The antifungal activity was tested against the fungus *Aspergillus niger* (ATCC 6275) and the yeast *Candida albicans* (ATCC 10231). Briefly, a 96-microwell plate was used for testing the antifungal activity at a concentration of the EO ranging from 4000 to 15.62 µg/mL, through two-fold serial dilution; the antifungal activity was reported as MIC. The final concentration of spores was 5 × 10^4^ spores/mL. The EO was dissolved in SDB with fungal inoculum to acquire the required concentrations. The maximum evaluated concentration was 4000 µg/mL; amphotericin B was used as the positive control, and DMSO as the negative control.

### 4.9. Evaluation of Antioxidant Capacity

#### 4.9.1. ABTS Radical Cation Scavenging Activity

The free radical scavenging capacity of the essential oil (EO) isolated from *M. ericifolia* was evaluated using the ABTS assay, also referred to as the Trolox Equivalent Antioxidant Capacity (TEAC) method [54]. This technique is based on the formation of the ABTS radical cation (ABTS•^+^) through an oxidation process. The assay was performed according to the procedure previously reported by Valarezo et al. (2021) [55], using a colorimetric method and a UV–Vis spectrophotometer (Genesys 10S UV–Vis, Thermo Fisher Scientific, Waltham, MA, USA).

The antioxidant activity of the EO was determined by monitoring the reduction of ABTS•^+^ at 734 nm. The antiradical capacity was expressed as the half-scavenging concentration (SC_50_). The highest EO concentration evaluated was 10,000 µg/mL. Trolox was used as the positive control, while methanol served as the negative control. The SC_50_ value, defined as the concentration required to achieve 50% inhibition, was calculated from the plot of percentage inhibition versus the logarithm (log_10_) of the EO concentration. Finally, the TEAC value was determined from the ratio between the SC_50_ of trolox (µM/mL) and the SC_50_ of the EO sample (g/mL).

#### 4.9.2. DPPH Radical Scavenging Activity

The free radical scavenging activity of the EO from *M. ericifolia* was also evaluated using the DPPH assay. In this method, the DPPH reagent generates the DPPH radical (DPPH^•^). The assay was conducted following the procedure described by Valarezo et al. (2021) [55]. The maximum evaluated concentration was 10,000 µg/mL. The equipment, as well as the negative and positive controls, was the same as that used in the ABTS assay, with the difference being that the antiradical capacity of the EO was assessed by measuring the reduction of DPPH^•^ at 515 nm.

### 4.10. Determination of Anticholinesterase Activity

Anticholinesterase activity was evaluated based on the method described by Ellman et al. [56], following the protocol detailed by Valarezo et al. (2021) [57]. The assay was performed using a microplate spectrophotometer (EPOCH 2, BioTek, Winooski, VT, USA). Briefly, reaction mixtures containing 50 mM Tris buffer (pH 8.0), acetylthiocholine, Ellman’s reagent (DTNB), and the EO at decreasing concentrations were pre-incubated at 25 °C for three minutes. The reaction was initiated by adding acetylcholinesterase, and its progression was monitored at 412 nm. The IC_50_ (half-inhibitory concentration) was determined using a non-linear regression model (normalized response/initial reaction rate vs. log Inhibitor-variable slope). Donepezil served as the positive control, while MeOH was used as the negative control.

### 4.11. Statistical Analysis

All experimental data were recorded in Microsoft Excel spreadsheets and subsequently processed using Minitab 17 (version 17.1.0, Minitab LLC., State College, PA, USA) to obtain descriptive statistics, including measures of central tendency and standard deviation. The antioxidant and anticholinesterase activity results were evaluated using GraphPad Prism (version 6.0, GraphPad Software Inc., San Diego, CA, USA).

All experimental procedures, including essential oil extraction, physicochemical characterization, the identification of EO constituents and enantiomeric analysis, were carried out in triplicate. In contrast, antioxidant evaluation, anticholinesterase assays, and antimicrobial tests were performed with nine replicates to ensure the reliability and reproducibility of the results.

## 5. Conclusions

The present study provides an integrated physicochemical, chemical, and stereochemical characterization of the essential oil obtained from *Melaleuca ericifolia* leaves grown under Ecuadorian conditions, together with a preliminary assessment of selected biological activities. The oil was characterized by a predominance of oxygenated monoterpenes, with 1,8-cineole as the major constituent. Importantly, the enantiomeric analysis provided a stereochemical fingerprint of selected chiral terpenes, adding a complementary level of characterization beyond conventional GC–MS compositional profiling. These data provide a useful baseline for future comparisons of the chemical and stereochemical variability of *M. ericifolia* essential oils.

Under the experimental conditions used, the essential oil showed limited biological activity. No antibacterial activity was detected against the tested Gram-positive and Gram-negative bacteria, while only weak antifungal activity was observed against *Candida albicans* and *Aspergillus niger*. Likewise, the values obtained in the ABTS and DPPH assays indicated weak direct radical-scavenging activity. Acetylcholinesterase inhibition was moderate and should be regarded as a preliminary screening result requiring further investigation.

The main contribution of this study lies in establishing a detailed chemical and stereochemical profile of *M. ericifolia* essential oil produced under Ecuadorian conditions. The findings also provide information on the chemical expression of an acclimatized exotic species growing under local environmental conditions and establish reference data for comparisons with oils obtained from the same species in other geographic regions.

Future research should therefore prioritize the evaluation of geographic, seasonal, phenological, and environmental effects on the chemical and enantiomeric profiles of *M. ericifolia* essential oil. The acetylcholinesterase-inhibitory activity observed here may also warrant targeted follow-up studies to determine the contribution of individual constituents and possible interactions among them. In addition, in vivo studies and complementary biological models, including anti-inflammatory and insecticidal assays, could provide a more comprehensive assessment of the potential applications of this essential oil.

## Figures and Tables

**Table 1:** Physical properties of the essential oil from leaves of *Melaleuca ericifolia.*

	*Melaleuca ericifolia* Leaf EO	
	Mean	SD
Density, *ρ* (g/cm^3^)	0.9221	0.0041
Refractive index, n^20^	1.4650	0.0001
Optical rotation, [*α*] (°)	+3.8	0.1
Color		
Subjective	Dark moderate yellow	
RGB color values	141, 122, 57	
CMYK color values	0%, 13%, 60%, 45%	
Hex Color Codes	#8D7A39	

SD: standard deviation.

**Table 2:** Chemical composition of the essential oil from leaves of *Melaleuca ericifolia.*

CN	RT	Compound	RIC	RIR	RA (%)	SD	CG	CF
1	9.03	*α*-Thujene	924	924	0.07	0.01	MH	C_10_H_16_
2	9.36	*α*-Pinene	932	932	4.97	0.17	MH	C_10_H_16_
3	10.12	Camphene	945	946	0.06	0.00	MH	C_10_H_16_
4	11.15	Sabinene	968	969	0.09	0.01	MH	C_10_H_16_
5	11.39	*β*-Pinene	973	974	3.71	0.17	MH	C_10_H_16_
6	11.88	Myrcene	987	988	0.87	0.06	MH	C_10_H_16_
7	12.61	*ρ*-Mentha-1(7),8-diene	1002	1003	0.11	0.01	MH	C_10_H_16_
8	13.69	*ρ*-Cymene	1018	1020	1.11	0.07	MH	C_10_H_14_
9	13.84	Limonene	1022	1024	11.23	0.16	MH	C_10_H_16_
10	14.09	1,8-Cineole	1027	1026	61.85	1.46	OM	C_10_H_18_O
11	14.64	(*Z*)-*β*-Ocimene	1033	1032	0.27	0.01	MH	C_10_H_16_
12	15.98	*γ*-Terpinene	1053	1054	0.06	0.00	MH	C_10_H_16_
13	18.03	Linalool	1096	1095	0.49	0.02	OM	C_10_H_18_O
14	18.65	*trans*-*ρ*-Mentha-2,8-dien-1-ol	1121	1119	0.05	0.00	OM	C_10_H_16_O
15	19.22	1-Undecyne	1123	1122	0.11	0.01	OC	C_11_H_20_
16	19.41	(Z)-Epoxy-ocimene	1129	1128	0.05	0.00	OM	C_10_H_16_O
17	19.58	*trans*-Pinocarveol	1136	1135	0.13	0.01	OM	C_10_H_16_O
18	20.02	Citronellal	1147	1148	0.09	0.02	OM	C_10_H_18_O
19	20.33	cis-dihydro-*β*-Terpineol	1154	1156	0.05	0.00	OM	C_10_H_20_O
20	21.07	*δ*-Terpineol	1164	1162	0.33	0.02	OM	C_10_H_18_O
21	21.47	Terpinen-4-ol	1176	1174	1.03	0.05	OM	C_10_H_18_O
22	21.90	*cis*-Pinocarveol	1184	1182	0.06	0.00	OM	C_10_H_16_O
23	22.28	*α*-Terpineol	1187	1186	7.99	0.10	OM	C_10_H_18_O
24	23.64	Citronellol	1225	1223	0.17	0.02	OM	C_10_H_20_O
25	24.73	Geraniol	1248	1249	0.10	0.01	OM	C_10_H_18_O
26	26.95	*ρ*-Cymen-7-ol	1291	1289	0.11	0.02	OM	C_10_H_14_O
27	27.92	Methyl geranate	1319	1322	0.18	0.02	OC	C_11_H_18_O_2_
28	29.52	Ethyl nerolate	1353	1351	0.06	0.01	OC	C_12_H_20_O_2_
29	31.25	Phenyl ethyl isobutanoate	1392	1393	0.44	0.04	OC	C_12_H_16_O_2_
30	32.10	(*Z*)-Caryophyllene	1410	1408	0.35	0.04	SH	C_15_H_24_
31	32.91	Aromadendrene	1437	1439	0.25	0.05	SH	C_15_H_24_
32	33.39	(*Z*)-*β*-Farnesene	1443	1440	0.39	0.05	SH	C_15_H_24_
33	33.85	9-*epi*-(*E*)-Caryophyllene	1462	1464	0.11	0.01	SH	C_15_H_24_
34	35.14	Viridiflorene	1494	1496	0.17	0.02	SH	C_15_H_24_
35	35.38	Bicyclogermacrene	1498	1500	0.07	0.00	SH	C_15_H_24_
36	36.92	*δ*-Amorphene	1511	1511	0.05	0.00	SH	C_15_H_24_
37	38.45	*epi*-Longipinanol	1561	1562	0.09	0.01	OS	C_15_H_26_O
38	38.55	Maaliol	1569	1566	0.07	0.01	OS	C_15_H_26_O
39	38.89	Spathulenol	1577	1577	0.48	0.04	OS	C_15_H_24_O
40	39.08	Caryophyllene oxide	1582	1582	0.32	0.03	OS	C_15_H_24_O
41	39.22	Globulol	1588	1590	0.65	0.04	OS	C_15_H_26_O
42	39.63	Cubeban-11-ol	1595	1595	0.32	0.08	OS	C_15_H_26_O
43	40.05	5-*epi*-7-*epi*-*α*-Eudesmol	1606	1607	0.24	0.13	OS	C_15_H_26_O
44	40.83	1-*epi*-Cubenol	1628	1627	0.05	0.00	OS	C_15_H_26_O
45	44.01	(2*E*,6*Z*)-Farnesol	1712	1714	0.11	0.02	OS	C_15_H_26_O
46	45.44	*α*-Bisabolol oxide A	1751	1748	0.11	0.01	OS	C_15_H_26_O_2_
47	50.37	(*E*)-Spiroether	1892	1890	0.05	0.00	OC	C_13_H_12_O_2_
Monoterpene hydrocarbons (MH)					22.55			
Oxygenated monoterpenes (OM)					72.50			
Sesquiterpene hydrocarbons (SH)					1.39			
Oxygenated sesquiterpenes (OS)					2.44			
Other compounds (OC)					0.84			
Total identified					99.72			

CF: chemical formula; CG: compound group; CN: compound number; RA: relative abundance; RIC: calculated retention indices; RIR: retention index based on literature; RT: retention time.

**Table 3:** Enantiomeric distribution of the essential oil from leaves of *Melaleuca ericifolia*.

RT	Enantiomers	RI	ED (%)	e.e. (%)
MEGA-DEX DET Beta column				
3.90	(+)-(1*R*,5*R*), (+)-(1*S*,5*S*), *α*-Pinene	934	81.83	63.66
4.23		948	18.17	
5.17	(+)-(1*R*,5*R*)-*β*-Pinene	993	4.93	90.14
5.22	(−)-(1*S*,5*S*)-*β*-Pinene	994	95.07	
7.65	(−)-(4*S*), (+)-(4*R*), Limonene	1058	100	-
21.02	(−)-(4*S*)-*α*-Terpineol	1299	23.38	53.23
21.43	(+)-(4*R*)-*α*-Terpineol	1305	76.62	
MEGA-DEX DAC Beta column				
24.22	(+)-(3*S*), (−)-(3*R*), Linalool	1290	75.57	51.15
24.37		1292	24.43	
30.27	(+)-(4*R*), (−)-(4*S*), *α*-Terpineol	1385	56.24	12.48
30.57		1489	43.76	

e.e.: enantiomeric excess; ED: enantiomeric distribution; RI: retention index; RT: retention time; -: presence of a single enantiomer.

**Table 4:** Antibacterial activity of essential oil from leaves of *Melaleuca ericifolia*.

Microorganism	*M. ericifolia* EO	Positive Control *	Negative Control
	MIC (µg/mL)		
Gram-negative bacilli			
*Campylobacter jejuni* (ATCC 33560)	NA	1.56	+
*Escherichia coli* O157:H7 (ATCC 43888)	NA	1.56	+
*Pseudomonas aeruginosa* (ATCC 10145)	NA	0.39	+
*Salmonella enterica* subs enterica serovar *Typhimurium* WDCM 00031, derived (ATCC 14028)	NA	0.39	+
Gram-positive bacillus			
*Listeria monocytogenes* (ATCC 19115)	NA	1.56	+
Gram-positive cocci			
*Enterococcus faecalis* (ATCC 19433)	NA	0.78	+
*Enterococcus faecium* (ATCC 27270)	NA	0.39	+
*Staphylococcus aureus* (ATCC 25923)	NA	0.39	+

*: Ciprofloxacin for Gram-positive bacillus and Gram-negative bacilli and ampicillin for Gram-positive cocci; MIC: minimum inhibitory concentration; +: normal growth; NA: no activity at the highest concentration tested (4000 µg/mL).

**Table 5:** Antimicrobial activity of essential oil from leaves of *Melaleuca ericifolia*.

Microorganism	*M. ericifolia* EO	Positive Control *	Negative Control
	MIC (µg/mL)		
Fungus			
*Aspergillus niger* (ATCC 6275)	4000	0.098	+
Yeast			
*Candida albicans* (ATTC 10231)	2000	0.098	+

*: Amphotericin B; MIC: minimum inhibitory concentration; +: normal growth.

**Table 6:** Antioxidant activity of essential oil from leaves of *Melaleuca ericifolia*.

Sample	ABTS	DPPH	TEAC
	SC_50_ ± SD (µg/mL) *		Mean ± SD (µM TE/g)
*Melaleuca ericifolia* EO	2001.1 ± 1.5	7657.5 ± 16.1	20.30 ± 2.3
Trolox	29.1 ± 1.1	35.5 ± 1.0	

ABTS: 2,2′-azinobis(3-ethylbenzothiazoline-6-sulfonic acid); DPPH: 2,2-diphenyl-1-picrylhydrazyl; TEAC: Trolox equivalent antioxidant capacity; SC_50_: half-maximal scavenging capacity; TE: trolox equivalent; *: µM for trolox; SD: standard deviation.

## Data Availability

Data are contained within the article.
